# Does Social Exclusion Improve Detection of Real and Fake Smiles? A Replication Study

**DOI:** 10.3389/fpsyg.2021.626087

**Published:** 2021-01-28

**Authors:** Simon Schindler, Martin Trede

**Affiliations:** Department of Psychology, University of Kassel, Kassel, Germany

**Keywords:** social exclusion, ostracism, need to belong, replication, smiles

## Abstract

Research on social exclusion suggests an increased attention of excluded persons to subtle social cues. In one study (*N* = 32), published in *Psychological Science*, Bernstein et al. (2008) provided evidence for this idea by showing that participants in the social exclusion condition were better in correctly categorizing a target person’s smile as real or fake. Although highly cited, this finding has never been directly replicated. The present study aimed to fill that gap. 201 participants (79.1% female) were randomly assigned to a social exclusion, social inclusion or control condition. Next, participants watched 20 videos of smiling persons and rated whether they show a real or a fake smile. In line with the original study, results showed that participants in the exclusion condition performed better than in the control condition. However, the performance did not differ between the exclusion and inclusion condition—although the pattern was in the predicted direction. In sum, the findings of our study increase rather than decrease confidence in the validity of the investigated idea, but results point to a substantially smaller effect.

## Introduction

Social exclusion threatens the basic need to belong and is therefore suggested to trigger adaptive mechanisms (Baumeister and Leary, 1995; Williams, 2009). One such mechanism refers to the activation of the social monitoring system (Pickett and Gardner, 2005) meaning searching the environment for information relevant to restoring need satisfaction and increase careful processing of that information. In other words, individuals are more attentive to signals and cues that indicate possible exclusion or rejection. In line with this idea, Pickett et al. (2004) found that people with a higher dispositional need for belonging are better at distinguishing facial expressions (angry, anxious, happy, and sad) or vocal tones (positive vs. negative). Gardner et al. (2000) could further show that excluded persons have a better memory for socially relevant information than non-excluded persons. More recently, Eck et al. (2020) showed that excluded individuals base their veracity judgments less on stereotypical non-verbal cues if message content is affiliation-relevant. These cases suggest that people show increased attention to social cues when they fear or experience rejection.

Bernstein et al. (2008) tested this idea by investigating how well people can distinguish between a real and a fake smile after social exclusion. Assuming an increased attention of excluded persons to subtle social cues and that facial expressions of emotion can act as such social cues, they expected that excluded people have an increased ability to distinguish between a real and fake smile. Research showed that Duchenne smiles (i.e., smiles that are formed by flexing facial muscles making the eyes’ corners wrinkle up with crow’s feet) were associated with self-reported pleasure and enjoyment and were generally rated more positively than non-Duchenne smiles (i.e., smiles that are formed with no contraction of the muscles of the corner of the eyes; Ekman et al., 1990; Gunnery and Ruben, 2016). In addition, the same brain regions that are activated when experiencing a positive emotion seem to be activated when forming a Duchenne smile, unlike other types of smiles (Ekman et al., 1990). In contrast, the non-Duchenne smile can be used to hide or mask negative emotions (Ekman et al., 1988). Results from Krumhuber et al. (2007) and Johnston et al. (2010) showed that people who showed a real, authentic smile were considered more trustworthy and positive compared to people who showed a fake smile. Furthermore, subjects were more likely to cooperate with people who showed a real smile (Johnston et al., 2010) and were more likely to choose them for a “trust game” (Krumhuber et al., 2007). Bernstein et al. (2008) concluded that distinguishing real from fake emotions seems especially important to socially excluded individuals to ensure that reaffiliation efforts are maximally distributed toward people displaying genuine affiliative cues. Therefore, directing resources toward an individual faking an affiliative display would likely be a costly error for socially rejected individuals, who already find themselves in a perilous situation.

According to their hypothesis, in one study (*N* = 32), Bernstein et al. (2008) showed that participants in the exclusion condition showed a significantly better discrimination ability (*M* = 1.88, *SD* = 0.62) than participants in the control condition (*M* = 1.05, *SD* = 0.56) and participants in the inclusion condition (*M* = 1.34, *SD* = 0.56). They found no significant differences between the control and inclusion conditions.

We believe that directly replicating this study is important for several reasons: first, the high estimated prevalence of questionable practices in past social psychological research asks for direct replication of prior studies (John et al., 2012; Simonsohn et al., 2014; Schindler et al., 2021)—especially when not preregistered and when having used small samples as in the present case (i.e., about 10 participants per cell). Second, the article of Bernstein et al. (2008) can be described as highly influential (more than 300 citations on google scholar). Third, the idea that social exclusion enhances attention to social cues has important theoretical implications for our basic understanding of human nature and the role of group affiliation. However, the evidence on this idea is based on a handful of studies. For example, Bernstein et al. (2010) *conceptually* replicated the findings of Bernstein et al. (2008) by showing that participants in the social exclusion condition indicated a greater preference to work with individuals displaying real (vs. fake) smiles. However, there has not been a direct replication. So, a direct replication of the study of Bernstein et al. (2008) makes a valuable contribution by increasing or decreasing confidence in this idea.

Parallel to the original study by Bernstein et al. (2008), we hypothesized that participants in the exclusion condition will show an enhanced ability to discriminate between real and fake smiles, compared to participants in the inclusion condition as well as participants in the control group. Going beyond the study of Bernstein et al. (2008), we included a measure of need to belong as a potential moderatoring variable (cf., Pickett et al., 2004).

## Method

### Ethics and Transparency Statements

The study was conducted in full accordance with the Ethical Guidelines of the German Association of Psychologists (DGPs) and the American Psychological Association (APA). Moreover, by the time the data were acquired it was also not required at Kassel University, nor at most other German universities to seek ethics approval for simple studies on personality and attitudes. The study exclusively makes use of anonymous questionnaires. No identifying information was obtained from participants. The participants were explicitly informed that the data are treated confidentially. Furthermore, they could withdraw from the study at any time. Participants answers and the effects on need threat revealed that psychological discomfort through thinking of being socially excluded was not particularly strong. Data, material, and the preregistration protocol of the study are available on the OSF^1^.

### Materials

#### Social Status Manipulation

Participants were randomly assigned to one of the three experimental conditions (exclusion vs. inclusion vs. control). In one essay task, participants were asked to write about a time they felt “rejected or excluded,” “accepted or included,” or “their morning yesterday.”

#### Visual Stimuli

The facial stimuli were those used in Bernstein et al. (2008) and were obtained from the BBC science and nature website (BBC – Science & Nature – Human Body and Mind – Spot The Fake Smile, 2015). Participants watched 20 videos (approximately 4 s each) one at a time, each depicting an individual with an initially neutral expression that shifted to a smiling expression, that then returned to a neutral expression (10 Duchenne and 10 non-Duchenne smiles). Thirteen men and seven women were depicted in the videos. The stimuli included three minority-group individuals. Removing data for these targets from analyses did not change any findings.

#### Need Threat

In the study of Bernstein et al. (2008), participants responded to a scale assessing the degree to which they felt a threat to their sense of belonging referring to the work of Williams et al. (2000). However, Williams and colleagues measured of belongingness toward other players in a cyberball game. It is obvious that these measures are not suitable for the exclusion manipulation of Bernstein et al. (2008). Unfortunately, Bernstein et al. (2008) did not report the wording of their used scale. Therefore, as a manipulation check for social status, we used items of the *Actual-Desired Need State* Scale (ADNS; Eck et al., 2017)—a measure consisting of twelve items referring to the difference between an actual and a desired state regarding four central needs: belonging (e.g., “I do not have as strong a sense of belonging as I would like”), self-esteem (e.g., “I have the feeling that others think worse of me than I would like”), control (e.g., “I have a feeling of having too little control over what is going on around me”), and meaningful existence (e.g., “I have a feeling of being less important at the moment than I wish”). The scale ranged from 1 (*not true at all*) to 7 (*completely true*). Reliability for threat to belonging was acceptable (α = 0.69) and higher for all twelve items (α = 0.89).

#### Need to Belong

As a potential moderator, need to belong was measured with the German version of Leary et al. (2013) *Need to Belong Scale* (NBS) by Hartung and Renner (2014). The scale consisted of ten items (e.g., “I have a strong need to belong”) and values ranged from 1 (*strongly disagree*) to 5 (*strongly agree*). Reliability was acceptable (α = 0.75).

### Procedure

The survey took about 13 min to complete and could be conducted with a laptop or smartphone. After agreeing to informed consent, participants were randomly assigned to one of the three experimental conditions. Next they answered the ADNS Items. Finally, participants watched each video in a random order and indicated, whether the smile was “genuine” or “fake” by choosing one of these options after each video. Upon completion of this task, participants responded to the NBS and demographic questions before being probed for suspicion or previous participation in a similar experiment and were thanked.

### Participants

In the study of Bernstein et al. (2008), the effect sizes of the two statistical contrasts were large with *d* = 1.41 (95% CI = [0.45, 2.36], for exclusion vs. control condition) and *d* = 0.91 (95% CI = [0.02, 1.81]; for exclusion vs. inclusion condition). The one-way analysis of variance (ANOVA) revealed an effect size of *d* = 1.25 with a *p* value of 0.009. As small sample studies are likely to overestimate the true effect and given the large confidence intervals of the found effects, we decided to assume a moderate effect of *d* = 0.5. An *a priori* power analysis for a one-way ANOVA (3 groups, alpha = 0.05, and *f* = 0.25) suggested including 207 participants to detect a significant effect with 90% power. Participants were randomly assigned to the three experimental conditions (exclusion vs. inclusion vs. control). That is, with about 70 participants per cell, we multiplied the original cell size by seven.

The data collection took place from the beginning of May until the end of July 2020. Participants were recruited mainly through social networks (e.g., Facebook) and personal contacts. The final sample consisted of 201 participants (159 female, *M*_*age*_ = 25.72, *SD* = 6.46, and range: 17–57; 73.1% students). Nineteen of the original 220 participants were excluded from the data analysis because they met the exclusion criteria specified in the pre-registration: eight participants were excluded because they did not give a meaningful answer in the essay task manipulation (i.e., any answer that could not be interpreted in a reasonable way and that could not be plausibly related to the questions in the broadest sense; e.g., “Can’t remember an event.”); two participants were excluded because they correctly recognized the background of the study; five participants were excluded because they reported technical problems playing the videos and were unable to complete the task properly; four participants were excluded because they had previously participated in a similar experiment to distinguish between real and fake smiles.

## Results

### Need Threat

To examine whether the social status manipulation affected fundamental needs, we conducted a one-way between-subjects ANOVA on the belonging threat. Although need for belonging was descriptively higher in the exclusion condition (*M* = 3.71, *SD* = 1.23) compared to the inclusion condition (*M* = 3.34, *SD* = 1.21) and the control condition (*M* = 3.53, *SD* = 1.20), the three conditions did not significantly differ from each other, *F*(2, 198) = 1.41, and *p* = 0.247. Simple effect analyses also revealed no significant differences, all *p*s > 0.095. There was further no significant effect of the manipulation on the whole ADNS scale, *F*(2, 198) = 1.05, and *p* = 0.352.

### Discrimination Scores

**FIGURE 1:**
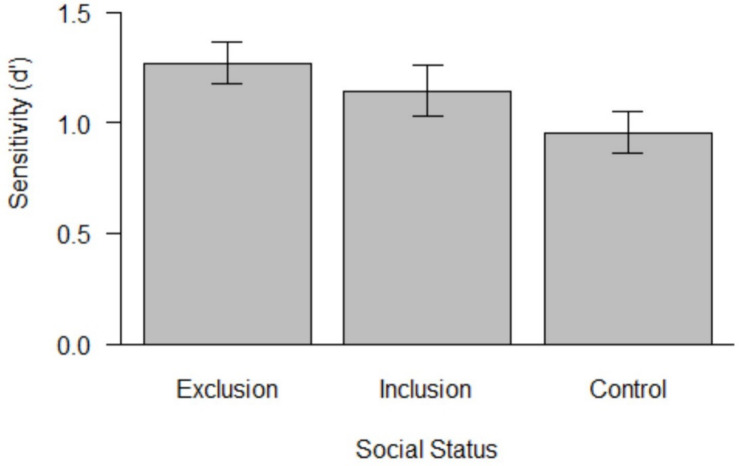
Mean ability to discriminate (sensitivity, *d’*) Duchenne and non-Duchenne smiles as a function of social status condition. Error bars indicate standard errors.

Parallel to Bernstein et al. (2008), we calculated *d’*, a signal detection measure examining the ability to discriminate stimuli—in this case, the ability to discriminate Duchenne smiles from non-Duchenne smiles. This measure simultaneously considers hits (correctly identifying a Duchenne smile as genuine) and false alarms (incorrectly identifying a non-Duchenne smile as genuine) in the calculation (Stanislaw and Todorov, 1999). The following scores were obtained (see Figure 1): *M*_*excl.*_ = 1.27 (*SD* = 0.77), *M*_*incl.*_ = 1.14 (*SD* = 0.87), and *M*_*contr*__*ol*_ = 0.95 (*SD* = 0.84). This basically replicates the descriptive pattern of Bernstein et al. (2008). The one-way ANOVA on *d’* was not significant, *F*(2,198) = 2.69, *p* = 0.070, and *d* = 0.33. A Bayesian analysis revealed a BF_10_ of 0.56, favoring the null hypothesis (Lee and Wagenmakers, 2013). Additional simple effects analyses showed that participants in the excluded condition scored significantly better compared to participants in the control condition, *M*_*diff*_ = 0.32, 95% CI *M* = [0.04, 0.59], *p* = 0.023, *d* = 0.32, but not compared to participants in the inclusion condition, *M*_*diff*_ = 0.13, 95% CI = [−0.17, 0.42], *p* = 0.394, and *d* = 0.12. Participants in the included condition did not differ significantly in their sensitivity measure from participants the control condition, *M*_*diff*_ = 0.19, 95% CI = [−0.09, 0.47], *p* = 0.190, and *d* = 0.18.

### Need to Belong

Need to belong was not significantly affected by the social status manipulation, *F*(2, 198) = 0.64, *p* = 0.531. We conducted a moderated regression on *d’* with the experimental conditions (as dummy coded variables with the exclusion condition as the reference category), need to belong as continuous predictor (*z*-transformed) and the interaction terms. The moderated regression again showed a significant main effect of the exclusion vs. control condition dummy variable, *b* = −0.33, *p* = 0.016, and no significant main effect of the inclusion vs. exclusion condition dummy variable, *b* = −0.14, and *p* = 0.362. There was no significant main effect of need to belong, *b* = 0.12, *p* = 0.213. Including the two interaction terms revealed no significant interaction effects, *b*_*i*__*ncl*_. = −0.06, *p* = 0.675, and *b*_*c*__*ontr*__*ol*_ = −0.026, *p* = 0.849.

## Discussion

The main goal of this study was to directly replicate the results of the study by Bernstein et al. (2008). The main hypothesis being tested was that being socially excluded enhances the ability to correctly distinguish between real and fake smiles. This was based on the assumption that excluded individuals should have a higher sensitivity to social cues. The original study showed the expected significant discrimination differences between the exclusion and inclusion condition as well as between the exclusion and neutral conditions. Both comparisons showed higher discrimination scores for participants in the exclusion condition. These results were only partially confirmed in the present study. While the descriptive pattern mirrored the findings of Bernstein et al. (2008), the ANOVA was not significant but only approached a significant level. Simple effect analyses revealed that participants in the exclusion condition were significantly better compared to participants in the control condition, but not compared to participants in the inclusion condition.

In contrast to Bernstein et al. (2008), there was no evidence for an effect of the social exclusion manipulation on need threat. Given that there is some support for the effect of the manipulation on the main dependent variable, an effect on self-reported need satisfaction may not be required (Sigall and Mills, 1998), especially because *unconscious* processes can be assumed that are difficult to assess with explicit measures (Eck et al., 2020). It is therefore questionable whether self-report measures on need threat are generally capable to capture the activation and operation of the social monitoring system.

Due to missing information in the original article, we used different items to assess need threat. These items referred to participants’ experience at the present moment, that is, after they thought and wrote about a respective event. This might be problematic—especially with autobiographic threat manipulations, because need threat represents negative feelings as a consequence of social exclusion. Thus, one could argue that need threat represents a dependent variable rather than a valid manipulation check (as labeled by Bernstein et al., 2008). As a more valid manipulation check, we coded participants’ written answers according to our three experimental conditions: being excluded, being included, neutral. In the exclusion condition, 98.5% of the answers were coded as “being excluded” and 1.5% as “neutral.” In the inclusion condition, all of the answers were coded as “being included.” In the neutral condition, 83.1% were coded as “neutral,” 15.6% as “being included” and 1.3% as “being excluded.” Accordingly, our manipulation can be regarded as successful. Interestingly, 56.1% of the answers in the inclusion condition also referred to some kind of uncertainty (which was then reduced by being included). Thus, the inclusion condition appears to be contaminated by thoughts about negative social events. The most adequate test for our hypothesis therefore refers to the comparison between the exclusion and the neutral condition—and this comparison revealed a significant result in favor of the hypothesis. Nevertheless, future research should apply stronger, validated manipulations that are based on an actually induced exclusion experience (e.g., cyberball; Williams et al., 2000) rather than remembering a past exclusion experience.

We further tested the moderation role of need to belong, because this variable was found to be positively related to distinguishing various facial expressions or vocal tones (Pickett et al., 2004). However, in our study, need to belong was unrelated to discrimination performance and also did not moderate effects of social status.

Bernstein et al. (2008) did not report specific sample characteristics but mentioned that their participants were undergraduates. This might indicate that participants were a little younger (about 20 years old) than in the present study (mean age of about 26 years). Interestingly, there is evidence for age-related changes in the effect of being socially excluded. Pharo et al. (2011), for example, found that adolescents and emerging adults experienced increased sensitivity to social exclusion (i.e., ostracism) relative to older participants (of about the age as in our study). This would provide another explanation for a lower effect size in our study compared to the study of Bernstein et al. (2008). At the same time, it seems reasonable to assume that social exclusion does always hurt—independent from the age—so, the role of age remains speculative.

Compared to the original study (*N* = 32), our study (*N* = 201) included nearly seven times more participants. We had 90% power to detect a moderate effect of *d* = 0.5. Given that the original study reported an effect of *d* = 1.25, our sample size seemed as a conservative choice. Nevertheless, it is important to note that effect sizes are decreased by error variance and there are plausible reasons why there was higher error variance in our study. First, we collected data online, thus, we had no control under which circumstances participants completed the study (via smartphone outside vs. at a PC at home). Given that the study was about recalling an experience of being socially rejected and on judging short videos, concentration and motivation might be lower in online studies adding error variance. Nevertheless, we chose to collect data online for several reasons: First, we could obtain larger sample sizes than by recruiting on campus. Second, online studies have become popular (Sassenberg and Ditrich, 2019) and it is important for future research to know if and how findings can be replicated. Last but not least, data collection in the lab was currently impossible due to the Corona pandemic.

This points to another potential source for error variance: context effects of the Corona pandemic. One might speculate that in these times concerns about being socially isolated are generally heightened leading to problems regarding a proper control condition. However, there is no strong evidence for this claim. First, we checked the answers in the social status manipulation for the words “Corona” or “pandemic” and there was only one participant (in the inclusion condition) who mentioned the word “Corona.” Second, data collection took place from May to July. In this time, the situation was highly under control in Germany: Kids were going back in school, people went on vacation, and patrons visited bars and restaurants. Open-air baths were accessible, and yet the number of infections remained quite stable at a low level, so that highly threatening information about the pandemic in Germany was rarely present in the news. Nevertheless, we cannot exclude that data collection during the pandemic did not add some error variance.

So far, only a few studies directly addressed the idea that social exclusion enhances attention to social cues. The present study makes an important contribution by directly replicating an influential small-*N* study of Bernstein et al. (2008). Overall, our findings increase rather than decrease confidence in the validity of the investigated idea, but results point to a substantially smaller effect.

## Data Availability Statement

The datasets presented in this study can be found in online repositories. The names of the repository/repositories and accession number(s) can be found below: https://osf.io/paedq/.

## Ethics Statement

Ethical review and approval was not required for the study on human participants in accordance with the local legislation and institutional requirements. The patients/participants provided their written informed consent to participate in this study.

## Author Contributions

Both authors listed have made a substantial, direct and intellectual contribution to the work, and approved it for publication.

## Conflict of Interest

The authors declare that the research was conducted in the absence of any commercial or financial relationships that could be construed as a potential conflict of interest.

## Footnotes

1 https://osf.io/paedq/

## References

[B1] Baumeister R. F. Leary M. R. (1995). The need to belong: desire for interpersonal attachments as a fundamental human motivation. *Psychol. Bull.* 117 497–529. 10.1037/0033-2909.117.3.497 7777651

[B2] BBC – Science & Nature – Human Body and Mind – Spot The Fake Smile (2015). *BBC - Science & Nature - Human Body and Mind - Spot The Fake Smile.* Available online at: http://www.bbc.co.uk/science/humanbody/mind/surveys/smiles/ (accessed February 10, 2015)

[B3] Bernstein M. J. Sacco D. F. Brown C. M. Young S. G. Claypool H. M. (2010). A preference for genuine smiles following social exclusion. *J. Exp. Soc. Psychol.* 46 196–199. 10.1016/j.jesp.2009.08.010

[B4] Bernstein M. J. Young S. G. Brown C. M. Sacco D. F. Claypool H. M. (2008). Adaptive responses to social exclusion: social rejection improves detection of real and fake smiles. *Psychol. Sci.* 19 981–983. 10.1111/j.1467-9280.2008.02187.x 19000206

[B5] Eck J. Schoel C. Greifeneder R. (2017). Belonging to a majority reduces the immediate need threat from ostracism in individuals with a high need to belong. *Eur. J. Soc. Psychol.* 47 273–288. 10.1002/ejsp.2233

[B6] Eck J. Schoel C. Reinhard M. A. Greifeneder R. (2020). When and why being ostracized affects veracity judgments. *Pers. Soc. Psychol. Bull.* 46 454–468. 10.1177/0146167219860135 31313631

[B7] Ekman P. Davidson R. J. Friesen W. V. (1990). The Duchenne smile: emotional expression and brain physiology. II. *J. Pers. Soc. Psychol.* 58 342–353. 10.1037/0022-3514.58.2.342 2319446

[B8] Ekman P. Friesen W. V. O’Sullivan M. (1988). Smiles when lying. *J. Pers. Soc. Psychol.* 54 414–420. 10.1037/0022-3514.54.3.414 3361418

[B9] Gardner W. L. Pickett C. L. Brewer M. B. (2000). Social exclusion and selective memory: How the need to belong influences memory for social events. *Pers. Soc. Psychol. Bull.* 26 486–496. 10.1177/0146167200266007

[B10] Gunnery S. D. Ruben M. A. (2016). Perceptions of Duchenne and non-Duchenne smiles: a meta-analysis. *Cogn. Emot.* 30 501–515. 10.1080/02699931.2015.1018817 25787714

[B11] Hartung F.-M. Renner B. (2014). The need to belong and the relationship between loneliness and health. *Zeitschrift Für Gesundheitspsychol.* 22 194–201. 10.1026/0943-8149/a000129 28847212

[B12] John K. J. Loewenstein G. Prelec D. (2012). Measuring the prevalence of questionable research practices with incentives for truth telling. *Perspect. Psychol. Sci.* 23 524–532. 10.1177/0956797611430953 22508865

[B13] Johnston L. Miles L. Macrae C. N. (2010). Why are you smiling at me? Social functions of enjoyment and non-enjoyment smiles. *Br. J. Soc. Psychol.* 49(Pt 1) 107–127. 10.1348/014466609X412476 19296878

[B14] Krumhuber E. Manstead A. S. R. Cosker D. Marshall D. Rosin P. L. Kappas A. (2007). Facial dynamics as indicators of trustworthiness and cooperative behavior. *Emotion* 7 730–735. 10.1037/1528-3542.7.4.730 18039040

[B15] Leary M. R. Kelly K. M. Cottrell C. A. Schreindorfer L. S. (2013). Construct validity of the need to belong scale: mapping the nomological network. *J. Pers. Assess.* 95 610–624. 10.1080/00223891.2013.819511 23905716

[B16] Lee M. D. Wagenmakers E.-J. (2013). *Bayesian Cognitive Modeling: A Practical Course.* Cambridge: Cambridge University Press.

[B17] Pharo H. Gross J. Richardson R. Hayne H. (2011). Age-related changes in the effect of ostracism. *Soc. Influence* 6 22–38. 10.1080/15534510.2010.525852

[B18] Pickett C. L. Gardner W. L. (2005). *The Social Monitoring System: Enhanced Sensitivity to Social Cues as an Adaptive Response to Social Exclusion.* New York, NY: Psychology Press.

[B19] Pickett C. L. Gardner W. L. Knowles M. (2004). Getting a cue: the need to belong and enhanced sensitivity to social cues. *Pers. Soc. Psychol. Bull.* 30 1095–1107. 10.1177/0146167203262085 15359014

[B20] Sassenberg K. Ditrich L. (2019). Research in social psychology has changed between 2011 and 2016: larger sample sizes, more self-report measures, and more online studies. *Adv. Methods Pract. Psychol. Sci.* 2 107–114. 10.1177/2515245919838781

[B21] Schindler S. Reinhardt N. Reinhard M.-A. (2021). Defending one’s worldviews under mortality salience – testing the validity of an established idea. *J. Exp. Soc. Psychol.* 93:104087 10.1016/j.jesp.2020.104087

[B22] Sigall H. Mills J. (1998). Measures of independent variables and mediators are useful in social psychology experiments: but are they necessary? *Pers. Soc. Psychol. Rev.* 2 218–226. 10.1207/s15327957pspr0203_5 15647156

[B23] Simonsohn U. Nelson L. D. Simmons J. P. (2014). P-curve: a key to the file-drawer. *J. Exp. Psychol. Gen.* 143 534–547. 10.1037/a0033242 23855496

[B24] Stanislaw H. Todorov N. (1999). Calculation of signal detection theory measures. *Behav. Res. Methods, Instrum. Comput.* 31 137–149. 10.3758/BF03207704 10495845

[B25] Williams K. D. (2009). “Ostracism: a temporal need-threat model,” in *Advances in Experimental Social Psychology*, Vol. 41 ed. Zanna M. P. (San Diego, CA: Elsevier Academic Press), 275–314.

[B26] Williams K. D. Cheung C. K. T. Choi W. (2000). Cyberostracism: effects of being ignored over the internet. *J. Pers. Soc. Psychol.* 79 748–762. 10.1037//0022-3514.79.5.748 11079239

